# Artificial Intelligence-Enabled Electrocardiography for Potassium Abnormality Detection and Estimation: A Systematic Review

**DOI:** 10.7759/cureus.113226

**Published:** 2026-07-23

**Authors:** Baldeep Kaur, Guntas Singh Gill, Kiranpreet Kaur, Ankur Chaudhary, Ashutosh Garg

**Affiliations:** 1 General Medicine, Dr. B.R. Ambedkar State Institute of Medical Sciences, SAS Nagar, IND; 2 Cardiology, Adesh Medical College and Hospital, Shahabad, IND; 3 Nephrology, Dr. B.R. Ambedkar State Institute of Medical Sciences, SAS Nagar, IND

**Keywords:** 12-lead ecg, ai ecg, artificial intelligence and machine learning, hyperkalemia, hyperkalemia induced ekg changes, potassium abnormality, serum potassium level

## Abstract

Laboratory measurement of serum, plasma, or whole-blood potassium remains the reference standard for diagnosing hyperkalemia and hypokalemia, yet results are not always immediately available when rapid clinical decisions are required. Artificial intelligence-enabled electrocardiography (AI-ECG) has been proposed as an early, non-invasive tool that may identify potassium abnormalities while laboratory confirmation is pending.

This systematic review evaluated adult studies published between 2016 and 2026 in which AI or machine-learning (ML) techniques were applied to ECGs to detect hyperkalemia or hypokalemia, classify potassium status, or estimate continuous potassium concentration, always against a paired laboratory potassium reference standard. We searched multiple biomedical, preprint, trial, and citation databases through June 18, 2026, following PRISMA-DTA, PRISMA 2020, and PRISMA-S reporting principles. Methodological quality was assessed with QUADAS-2.

Of 366 records assessed for eligibility, 33 peer-reviewed primary AI/ML studies were included. Hyperkalemia detection dominated the evidence base, with many externally or internally validated studies reporting area under the receiver operating characteristic curve (AUROC) in the high 0.8 to mid-0.9 range, whereas evidence for hypokalemia detection, categorical potassium-status classification, and continuous potassium estimation remained overlapping and more heterogeneous. Although reported performance was often encouraging, overall risk of bias was low in only four studies, unclear in 23, and high in six. Considerable heterogeneity in potassium thresholds, ECG modalities, validation strategies, reporting units, and performance metrics precluded meta-analysis.

Overall, the available evidence suggests that AI-ECG is a promising investigational adjunct for triage, monitoring, and clinical decision support, primarily serving as a rule-out tool rather than a replacement for laboratory potassium testing. Its clinical utility may be particularly relevant in higher-prevalence populations, such as patients with chronic kidney disease or those receiving dialysis, where positive predictive value and monitoring yield may be more favorable. Prospective external validation, calibration, subgroup reporting, reproducibility, and workflow-impact studies are needed before routine clinical implementation.

## Introduction and background

Hyperkalemia and hypokalemia are common electrolyte disturbances encountered across emergency, inpatient, intensive care, dialysis, and ambulatory settings. They arise from kidney disease, endocrine disorders, acute illnesses, or medications. Severe potassium abnormalities can precipitate life-threatening arrhythmias requiring urgent treatment [1]. Timely recognition is therefore essential. Laboratory measurement of serum, plasma, or whole-blood potassium remains the diagnostic reference standard and continues to guide treatment decisions [1].

The electrocardiogram (ECG) has long been used to look for potassium-related changes, but its conventional findings are an unreliable substitute for laboratory potassium measurement. Classic features such as peaked T waves or QRS widening are inconsistently present and are influenced by baseline cardiac disease, medications, conduction abnormalities, and the severity and timing of the disturbance [2]. In clinical workflows, an ECG is often recorded before the potassium result becomes available, which has motivated interest in ECG-based tools that could prioritize testing or monitoring without waiting for the laboratory.

Artificial intelligence-enabled ECG (AI-ECG) refers to AI/machine-learning (ML) methods that learn or classify patterns from ECG data rather than relying on rule-based ECG criteria or conventional statistical regression. Recent advances in deep learning have enabled AI models to identify subtle ECG patterns that are not readily discernible by visual interpretation, creating opportunities for earlier detection of electrolyte abnormalities. These approaches have been developed using diverse ECG inputs: 12-lead, reduced-lead, single-lead, wearable, monitor-derived, and image-based recordings - and applied to four distinct tasks: detecting hyperkalemia, detecting hypokalemia, classifying potassium status into categories, and estimating a continuous potassium concentration [2-4].

Reported accuracy alone does not establish that such a model is ready for clinical use. A reported area under the receiver operating characteristic curve (AUROC), which summarizes discrimination across classification thresholds, or estimation error is difficult to interpret in isolation. Clinical utility depends on the potassium threshold, patient sampling, external validation (evaluation in an independent population beyond the development dataset), calibration (agreement between predicted and observed outcomes), and whether performance is maintained in individual patients under routine clinical conditions. Across the literature, these design features vary substantially, which makes it difficult to judge whether reported performance reflects transportable clinical accuracy or favorable development conditions.

Although narrative and modeling reviews have examined the conventional electrocardiographic changes associated with potassium disturbances [2], and a recent non-peer-reviewed preprint systematically reviewed AI-ECG across multiple electrolyte abnormalities while meta-analyzing potassium detection [5], our literature search did not identify any peer-reviewed systematic review specifically evaluating AI/ML-enabled ECG across the full spectrum of potassium-related applications. Existing syntheses have not jointly examined categorical potassium classification and continuous potassium estimation alongside detection, nor have they explicitly considered model lineage, i.e., relationships between successive versions of AI models and shared datasets, both of which may influence interpretation of the evidence.

This systematic review therefore addressed a single question: across the full spectrum of potassium-related tasks, how accurate and how clinically ready is AI-ECG when tested against a paired laboratory potassium measurement as the reference standard? We evaluated adult human evidence across four clinically relevant potassium tasks - hyperkalemia detection, hypokalemia detection, categorical potassium-status classification, and continuous potassium estimation. Particular attention was given to validation maturity (progression from model development through internal, external, prospective, and implementation evaluation), risk of bias, evidence independence, and the feasibility of quantitative synthesis.

## Review

Methodology

Design and Reporting Standards

This diagnostic-performance test systematic review was conducted and reported in accordance with PRISMA-DTA and PRISMA 2020 statements, with PRISMA-S used to guide literature search reporting [6-8]. Methodological quality and applicability were assessed using QUADAS-2 [9]. The review was not prospectively registered.

Eligibility Criteria (PICOS Framework)

Eligibility criteria followed the PICOS framework and is shown in Table 1.

**Table 1:** Eligibility criteria PICOS: Population, Intervention, Comparator, Outcomes, and Study Design; ECG: electrocardiography; AI: artificial intelligence; ML: machine learning; AUROC: area under the receiver operating characteristic curve

Component	Description
Population (P)	Adult human participants with electrocardiographic data paired with a laboratory potassium measurement.
Index test (I)	Artificial intelligence- or machine learning-based ECG methods, including deep learning, neural networks, conventional machine-learning algorithms, and previously developed AI-enabled ECG models applied to 12-lead, reduced-lead, single-lead, wearable, monitor-derived, image-based, or feature-derived ECG data.
Comparator / reference standard (C)	Reference standard: Laboratory measurement of serum, plasma, blood, or whole-blood potassium obtained in temporal pairing with the ECG (required for every included study). Optional comparator: Conventional ECG interpretation, clinician assessment, or point-of-care testing, when a study reported one alongside the AI/ML index test.
Outcomes (O)	Hyperkalemia detection, hypokalemia detection, categorical potassium-status classification, or continuous potassium concentration estimation, with extractable diagnostic accuracy or regression performance metrics (e.g., AUROC, sensitivity, specificity, calibration, mean absolute error, or correlation).
Study design (S)	Peer-reviewed retrospective or prospective primary studies evaluating diagnostic accuracy, prediction models, external validation, monitoring, or clinical implementation of AI/ML ECG methods for potassium assessment.

We included peer-reviewed journal articles and full-length conference proceedings papers for which a scientific-review process could be documented. Eligible studies were published between 2016 and 2026 and involved adult participants in whom AI/ML-based ECG methods were applied to potassium-specific outcomes, with extractable diagnostic or model-performance results against a paired laboratory potassium measurement as the reference standard. Other reports not falling into above criteria were excluded from the primary evidence synthesis.

Reviews, editorials, commentaries, protocols, and registry records without eligible peer-reviewed diagnostic data were excluded from the primary evidence set. We also excluded pediatric-only and non-human studies; in vitro or simulation-only studies; records outside the 2016-2026 eligibility window; non-ECG studies; studies without potassium diagnostic or estimation target; ECG studies that relied on conventional interpretation, rule-based criteria, or standard statistical analysis rather than a clearly defined AI/ML model; studies lacking paired laboratory potassium measurements, and studies without extractable potassium-specific performance outcomes. Related non-primary records were retained separately, only to clarify publication lineage, dataset overlap, trial or preprint status, and competing-review context. Paywall status alone was not used as a reason for exclusion.

Information Sources and Search Strategy

We conducted the principal formal searches in PubMed/MEDLINE, Europe PMC, ClinicalTrials.gov, the Cochrane Library/CENTRAL, medRxiv, OpenAlex, and Semantic Scholar. Crossref was used for DOI and bibliographic verification rather than as a primary literature-discovery source. Backward and forward citation chasing and targeted checks of arXiv, publisher pages, repositories, and engineering metadata sources, including DBLP and IEEE metadata or DOI pages, were used to identify related publications and to clarify publication status, bibliographic details, dataset overlap, and model lineage. These activities were record-specific supplementary verification procedures rather than comprehensive formal database searches. We did not directly search Scopus, Web of Science, Embase, or IEEE Xplore; the potential effect of this is considered in the limitations. Preliminary scoping searches began in April 2026; the structured, source-specific searches were conducted in May 2026 and expanded and reconciled through June 2026, with a final update check on June 18, 2026. No publication-date or language restriction was applied at the search stage. The included peer-reviewed primary studies were published between 2016 and 2026. Appendix 1 summarizes the information sources and supplementary verification procedures, while Appendix 2 presents the principal formal source-specific queries.

Study Selection Process

Records were deduplicated using DOI, PMID, PMCID, registry identifiers, arXiv identifiers, stable URLs, and normalized titles. Title and abstract screening used predefined eligibility criteria, with screening decisions independently verified by two reviewers. Records considered potentially eligible or of uncertain relevance advanced to full-text eligibility assessment. Near-miss records combining ECG, potassium or electrolyte, and AI/ML or signal-processing concepts underwent additional review before final classification as primary or non-primary evidence. Full-text eligibility decisions were reviewed collaboratively by the author team, and uncertainties were resolved through discussion and consensus. Each excluded record was assigned a controlled primary exclusion reason. arXiv identifiers were included in deduplication because preprint records encountered during targeted arXiv checks, citation tracing, and publication-status verification were retained to link preprints with related peer-reviewed reports.

Data Extraction

A structured form was used to extract bibliographic details, study characteristics, ECG source, reference standard, potassium threshold, model characteristics, validation design, performance metrics, calibration, clinical utility, funding, conflicts of interest, and dataset overlap. Initial data extraction was performed by designated reviewers and independently verified by additional members of the review team against the original articles, supplementary materials, and bibliographic metadata. Any discrepancies were resolved through discussion and consensus among the authors. Classification metrics were extracted and analysed separately from continuous estimation metrics, and any value derived from figures or confusion matrices was explicitly identified as derived. Author-reported confidence intervals were extracted where available; no new uncertainty intervals were calculated. Overlapping cohorts and related model lineages were mapped before evidence synthesis.

Quality Assessment and Risk of Bias

Risk of bias and applicability were assessed using QUADAS-2 [9]. Risk of bias was evaluated across the four QUADAS-2 domains-patient selection, index test, reference standard, and flow and timing-while applicability concerns were assessed across the first three domains, consistent with the tool’s structure. Initial QUADAS-2 assessments were performed by designated reviewers and independently reviewed by additional members of the review team. Any discrepancies or uncertain judgments were resolved through discussion and consensus among the authors following re-examination of the original reports. Given that the index tests comprised AI/ML models, additional AI-specific considerations were documented, including validation design, calibration, explainability, transparency, deployment readiness, dataset overlap, and the potential for optimism bias. Tailored signaling questions and final study-level judgments were recorded for all included studies.

Data Synthesis

We conducted a structured narrative synthesis, grouping studies according to potassium target, potassium threshold, ECG modality, clinical setting, validation maturity, unit of analysis, performance metric, risk of bias, and dataset overlap. Before deciding whether quantitative synthesis was appropriate, the review team applied the following comparability criteria: the same target condition; a comparable potassium threshold; a comparable ECG modality and input format; a comparable clinical setting; a comparable validation design and unit of analysis; cohort and model independence, with no shared dataset or model lineage; and sufficient reported diagnostic-accuracy data to reconstruct a 2 × 2 contingency table or an operating point with a measure of uncertainty. Studies were considered eligible for pooling only if all of these criteria were met. Otherwise, findings were synthesized narratively without quantitative pooling. Study-level estimates are reported with 95% confidence intervals where the primary studies provided them.

Results

Study Selection Process

**Figure 1:**
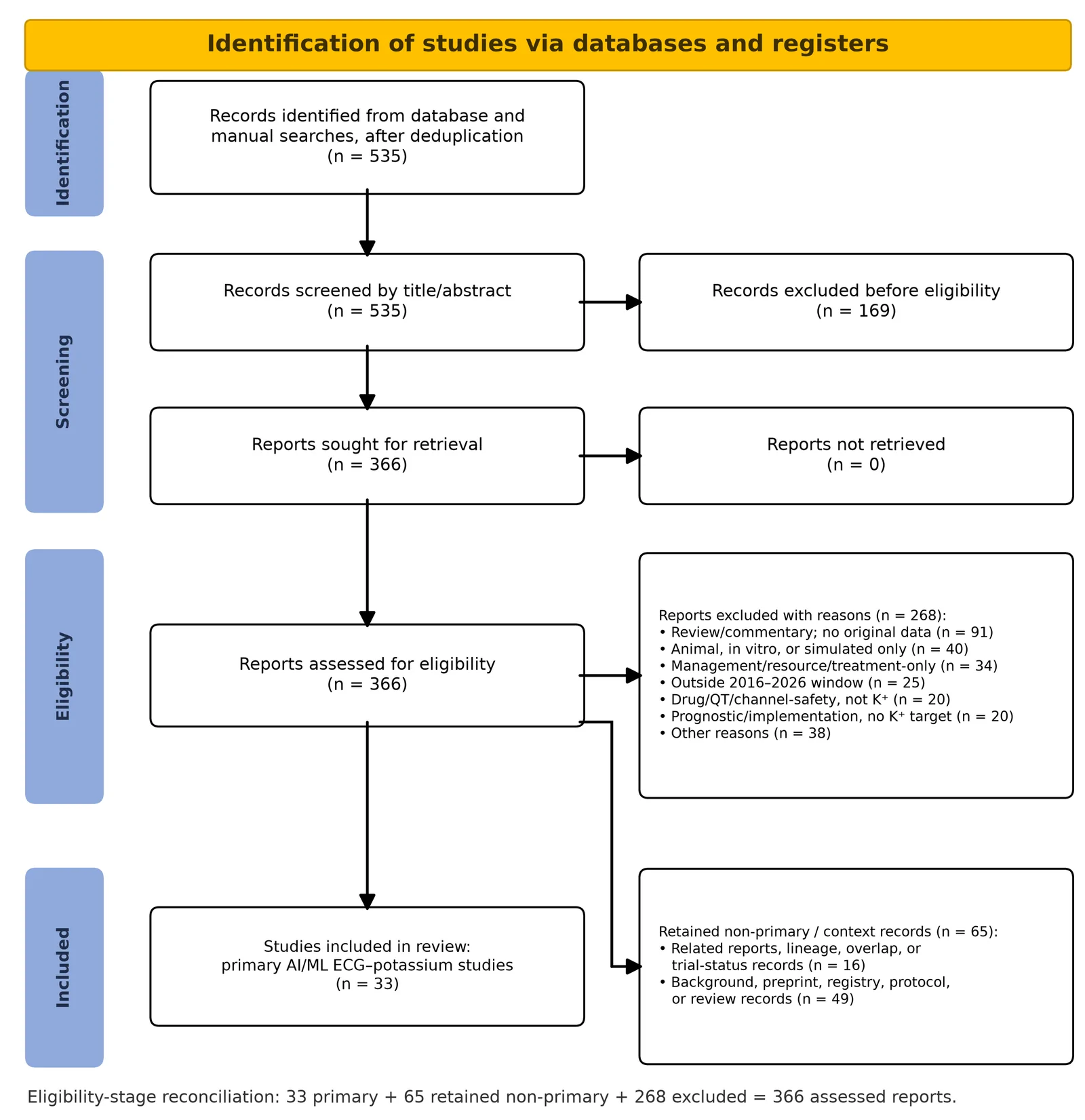
PRISMA-based study selection flow diagram Study selection for the present systematic review. The diagram begins after deduplication. PRISMA: Preferred Reporting Items for Systematic Reviews and Meta-Analyses; AI/ML: artificial intelligence/machine learning; ECG: electrocardiography

Of 366 records assessed for eligibility, 33 peer-reviewed primary AI/ML studies were included [3,4,10-40]. An additional 65 non-primary records were retained separately to document publication lineage, trial status, preprints, competing reviews, signal-processing or statistical context, and dataset overlap, while 268 records were excluded from the primary evidence set. The PRISMA flow diagram of study selection, including categorical reasons for exclusion are shown in Figure 1.

Study Characteristics

**Figure 2:**
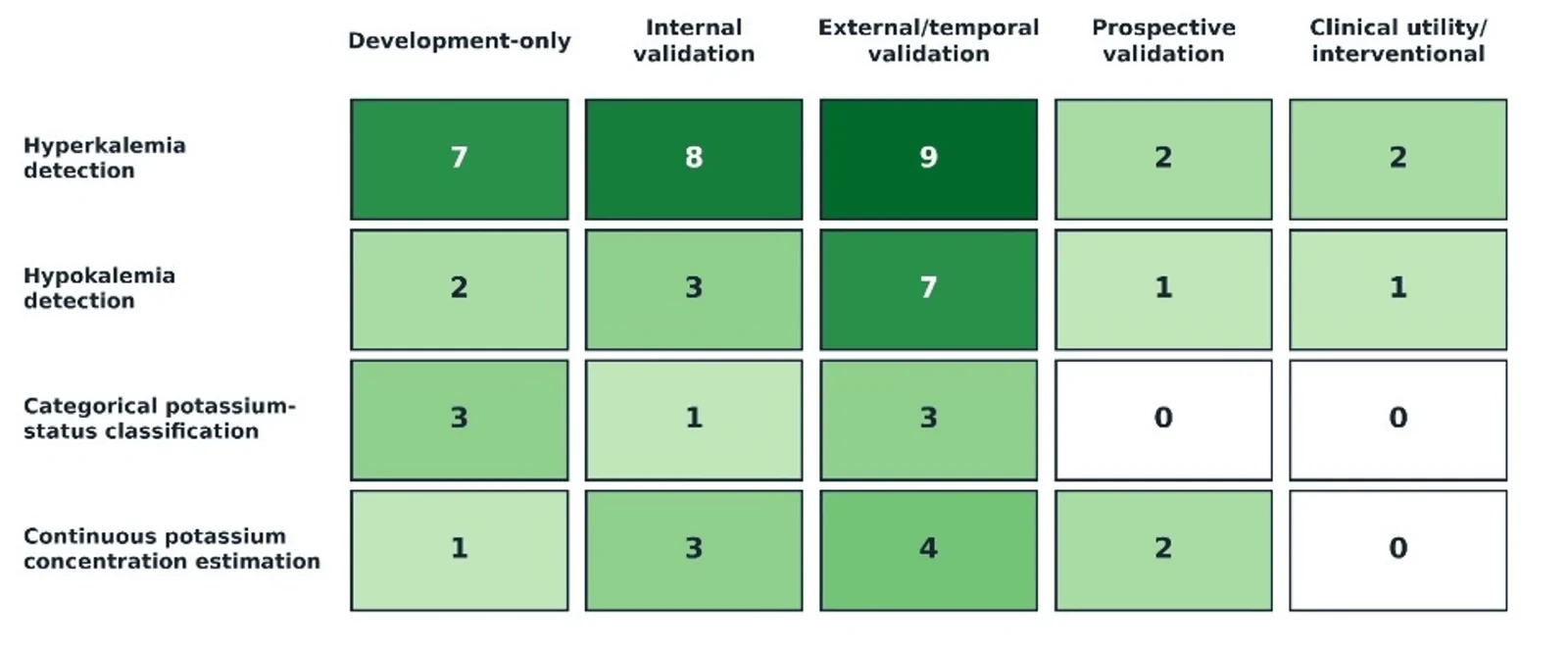
Evidence map of included primary AI/ML studies by potassium target and validation maturity Based on data extracted from the included primary AI/ML studies [3,4,10-40]. Cell shading indicates the number of primary studies in each cell. Because individual studies could address more than one potassium target, cell counts exceed the 33 included primary AI/ML studies. AI/ML: artificial intelligence/machine learning; ECG: electrocardiography

The 33 included primary AI/ML studies evaluated four AI-ECG tasks. Because many studies addressed more than one task, these categories were not mutually exclusive. Hyperkalemia detection was most common (28 studies), followed by hypokalemia detection (14), continuous potassium estimation (10), and categorical potassium-status classification (seven); 11 studies addressed both hyperkalemia and hypokalemia, and five also estimated other electrolytes such as sodium or calcium. Studies used diverse ECG inputs, including 12-lead and multi-lead recordings, reduced-lead and single-lead recordings, smartwatch and wearable signals, monitor-derived recordings, morphology- or feature-based inputs, and image-, beat-, or segment-level representations. Cohorts spanned emergency departments, dialysis units, intensive care, general inpatient settings, and remote monitoring, and several studies used large institutional ECG repositories. Figure 2 illustrates the distribution of studies by potassium target and validation maturity, while Table 2 summarizes the evidence base by potassium target.

**Table 2:** Evidence summary by potassium target Data synthesized from the included primary AI/ML studies [3,4,10-40]. Targets are not mutually exclusive. Eleven studies addressed both hyperkalemia and hypokalemia, several continuous-estimation studies also reported detection outcomes, and five studies evaluated potassium alongside other electrolytes. Consequently, study counts exceed the 33 included primary AI/ML studies. AI/ML: artificial intelligence/machine learning; AUROC: area under the receiver operating characteristic curve; ECG: electrocardiography

Potassium target	Studies (n)	Typical study characteristics	Representative findings	Key findings
Hyperkalemia detection	28	Common thresholds ranged from K+ >5.0 to >=6.5 mmol/L; most studies used binary detection, although some multiclass models included a hyperkalemia class.	Reduced-lead, single-lead, 12-lead, wearable, monitor-derived, and image-based ECG models were evaluated. Several studies reported AUROCs in the high 0.8 to mid-0.9 range.	Thresholds, ECG-to-laboratory timing, study populations, and validation designs varied substantially across studies.
Hypokalemia detection	14	Usually K+ <3.5 mmol/L; one study of thyrotoxic periodic paralysis used K+ <=3.0 mEq/L [34].	Most studies evaluated hypokalemia within combined dyskalemia or electrolyte models rather than dedicated hypokalemia models.	External validation was less frequently reported than for hyperkalemia studies.
Categorical potassium-status classification	7	Multiclass potassium categories or ordered potassium-status labels.	Deep-learning, neuro-fuzzy, wavelet/deep-learning, and semi-supervised approaches were reported.	Class definitions and performance metrics varied across studies.
Continuous potassium estimation	10	Continuous estimation (regression); study- defined thresholds	Reported mean absolute error ranged from approximately 0.26-0.53 mmol/L (where available).	Regression outcomes were reported separately from diagnostic accuracy metrics.

Detailed study characteristics and representative performance metrics for all 33 included primary AI/ML studies are provided in Appendix 3.

Evidence by Potassium Target

Hyperkalemia detection was the largest evidence group and varied in potassium threshold (commonly 5.0-6.5 mmol/L), study population, ECG modality, and validation design. Hypokalemia detection was reported by 14 studies, although most detected hypokalemia within a combined dyskalemia model that also addressed hyperkalemia. Only two studies evaluated hypokalemia as a dedicated screening task [35,36], whereas one focused on thyrotoxic periodic paralysis [34]. Seven studies evaluated categorical potassium-status classification, and 10 reported continuous potassium estimation using regression metrics such as mean absolute error, root mean squared error, and correlation. Five studies also evaluated potassium alongside other electrolytes. Detailed study characteristics are provided in Appendix 3.

Validation Maturity and Clinical Utility

Validation maturity varied across the evidence base: seven studies were development-only, 11 used internal validation, 10 used external or temporal validation, three included prospective validation, and two provided clinical monitoring, utility, or interventional evidence [26,28]. One study evaluated serial monitoring during treatment of severe hyperkalemia [26], whereas another reported the only identified pragmatic randomized trial of an AI-ECG alert [28]. The randomized trial found no significant overall increase in hyperkalemia- or hypokalemia-related treatment within three hours, although benefit was observed in the subgroup flagged as hyperkalemic [28].

Diagnostic and Model Performance

Performance reporting was heterogeneous. Binary and categorical studies reported AUROC, sensitivity, specificity, predictive values, accuracy, F1 score, or partial confusion-matrix data, while continuous-estimation studies reported regression error and correlation. Among hyperkalemia studies with internal or external validation, AUROCs were frequently in the high 0.8 to mid 0.9 range [3,10,13,18,23,24,31,33], and AI/ML models performing continuous potassium estimation reported mean absolute errors around 0.26-0.53 mmol/L in selected cohorts [4,13,23,37,39]. Study-level performance for each included study, with classification and continuous-estimation metrics shown separately, is tabulated in Appendix 3. 

Risk of Bias and Applicability

Overall risk of bias was low in four studies, unclear in 23, and high in six; overall applicability concern was low in 18 studies, unclear in 10, and high in five. Recurring concerns included case-control enrichment, artificial class balancing, incompletely reported ECG-to-laboratory timing, unclear train-test split integrity, limited threshold prespecification, sparse calibration reporting, limited reproducibility, and incomplete paired diagnostic data. Domain-level QUADAS-2 judgments are summarized in Table 3.

**Table 3:** QUADAS-2 risk-of-bias and applicability assessment Values represent the 33 included primary AI/ML studies [3,4,10-40]. QUADAS-2: Quality Assessment of Diagnostic Accuracy Studies, version 2; AI: artificial intelligence; ML: machine learning

Domain	Low	Unclear	High	Not applicable	Common findings
Patient selection: risk of bias	14	13	6	0	Case-control enrichment and sampling restrictions were common concerns.
Index test: risk of bias	7	25	1	0	Thresholding, model transparency, split integrity, and calibration reporting were often incomplete.
Reference standard: risk of bias	27	6	0	0	Laboratory potassium was generally appropriate, but specimen and timing details varied.
Flow and timing: risk of bias	9	24	0	0	Electrocardiography-laboratory timing and missing data were frequent reporting limitations.
Patient selection: applicability	21	7	5	0	Some populations were narrow or enriched compared with general clinical use.
Index test: applicability	32	1	0	0	Most index tests matched the strict AI/ML review question, with some engineering or representation-level caveats.
Reference standard: applicability	27	6	0	0	Reference-standard applicability was usually low concern.
Overall risk of bias	4	23	6	0	Overall risk of bias was rated as unclear in most studies.
Overall applicability concern	18	10	5	0	Applicability was mostly low concern but not uniformly so.

One included study carried an editorial expression of concern relating to data availability [18,41]. The study was retained in the review and considered during risk-of-bias assessment and sensitivity checks of the pooling decision rather than being excluded automatically.

Meta-Analysis Feasibility

No clinically coherent, independent group of studies met the comparability criteria for quantitative synthesis. Even within the most plausible candidate subgroup - hyperkalemia detection near K+ ≥5.5 mmol/L - studies differed in setting, ECG modality or input format, validation design, operating-point reporting, and cohort or model independence. The principal barriers were heterogeneous potassium targets and thresholds, varied ECG modalities and clinical settings, differing validation designs and reporting units, dataset overlap or shared model lineage, and incomplete paired diagnostic data. Related ECG model families, competition-derived subsets, and retained signal-processing lineage records were therefore not treated as independent validations, and these overlaps were assessed before synthesis. Sensitivity checks of the pooling decision did not alter this conclusion. Repeating the comparability assessment after restricting the evidence to author-reported metrics, or after excluding studies at high risk of bias, the study carrying an editorial expression of concern, or development-only studies, still yielded no clinically comparable and independent group suitable for pooling. Although a previous preprint meta-analysis pooled potassium-detection studies across institutions [5], our broader review scope together with the overlap- and lineage-independence assessment supported a structured narrative synthesis rather than quantitative pooling. Consequently, no pooled estimates, summary sensitivity, specificity, AUROC, bivariate model, HSROC model, forest plot, or summary ROC curve were generated. 

Discussion

This systematic review synthesized evidence from 33 peer-reviewed primary AI/ML studies evaluating AI-ECG for hyperkalemia detection, hypokalemia detection, categorical potassium-status classification, and continuous potassium estimation against a paired laboratory measurement as the reference standard [3,4,10-40]. To our knowledge, this is one of the first peer-reviewed systematic reviews to evaluate all four potassium-related AI-ECG tasks, while also appraising validation maturity and the independence of datasets and model lineage across studies. Three findings stand out. First, the evidence base is dominated by hyperkalemia detection, with hypokalemia detection, multiclass classification, and continuous potassium estimation represented by smaller and more heterogeneous subsets. Second, reported diagnostic performance was frequently high, but most evidence came from retrospective development or internal validation, with a smaller subset reaching external or temporal validation and only a few studies progressing to prospective or interventional evaluation. Third, and most importantly for clinical practice, among binary detection studies reporting predictive values, negative predictive values were generally high while positive predictive values were often low, indicating that current AI-ECG functions primarily as a rule-out and triage tool rather than a confirmatory test. Taken together, these findings position AI-ECG as a promising but still investigational adjunct to, rather than a replacement for, laboratory potassium measurement.

Diagnostic and Estimation Performance in Clinical Context

Overall diagnostic performance was encouraging. Among hyperkalemia studies with internal or external validation, AUROCs were frequently in the high 0.8 to mid 0.9 range [3,10,13,18,23,24,31,33], and single-lead or wearable implementations extended detection beyond the conventional 12-lead ECG [12,23,25]. However, confidence in these estimates varied according to validation maturity, with externally or prospectively validated cohorts providing the most clinically applicable evidence, whereas findings from smaller, engineering-oriented, or class-balanced development studies were less readily generalizable.

From a clinical perspective, predictive values are more informative because they depend on disease prevalence in the population being tested. In unselected populations, positive predictive value remained low despite good discrimination - for example, 1.4% with a corresponding negative predictive value of 99.9% [10], 3% and 99.8% in the emergency department, and 13.2% and 99.4% in intensive care [24,27]. Thus, in the populations and at the thresholds studied, a negative AI-ECG result may assist in ruling out dangerous hyperkalemia, whereas a positive result should prompt confirmatory laboratory testing rather than establish the diagnosis. As expected, positive predictive value increased in higher-prevalence populations, reaching approximately 15% in the emergency department and 29% in intensive care among patients with advanced chronic kidney disease and an estimated glomerular filtration rate of <30 mL/min [24]. Similarly, several models were developed specifically in dialysis and end-stage renal disease populations, where hyperkalemia is more prevalent [17,23,25,26]. Models performing continuous potassium estimation reported mean absolute errors of approximately 0.26-0.53 mmol/L [4,13,23,37,39]. These error estimates suggest possible utility for trend monitoring, but the included studies did not establish a consistent, prospectively validated clinical-acceptability margin. Errors of this magnitude may be clinically important near treatment thresholds; continuous AI-ECG estimates should therefore not be interpreted as exact potassium concentrations or used to direct treatment without laboratory confirmation. Diagnostic performance for hypokalemia was generally lower than for hyperkalemia, with AUROCs frequently below 0.8, particularly when hypokalemia was evaluated within combined dyskalemia models or using single-lead ECGs [15,35]. This likely reflects the subtler and less specific electrocardiographic manifestations of hypokalemia, such as T-wave flattening and U waves, which provide weaker signals for AI models to detect. Likewise, multiclass potassium-status classification should not be equated with binary detection of clinically significant dyskalemia, as accurate classification across ordered categories may still fail to identify patients at the clinically important extremes [4,11,13,22,30,37,39].

Comparison With Conventional ECG Reading and Point-of-Care Testing

The clinical rationale for AI-ECG lies in the limitations of conventional electrocardiographic interpretation. Classical ECG features of dyskalemia, such as peaked T waves and QRS widening, are inconsistently present and correlate poorly with the measured potassium concentration [2]. This was reflected in the included studies: when a smartphone-based AI-ECG analyzer was compared with board-certified emergency physicians for hyperkalemia detection, the model achieved an AUROC of approximately 0.90 compared with 0.66 for physician consensus, with physician sensitivity of only about 20% [16]. These findings suggest that AI models can identify electrocardiographic patterns that are not readily recognized by visual interpretation alone.

The more relevant bedside comparator, however, is point-of-care potassium testing. In one critical-care study, AI-ECG was slightly less accurate than point-of-care testing obtained from arterial blood gas analysis (AUROC 0.884 vs. 0.933), although both became available approximately 35 minutes before the central laboratory result [27]. AI-ECG should therefore be viewed as complementary rather than competitive; where point-of-care testing is available, it remains the preferred diagnostic approach, whereas AI-ECG offers an immediate, non-invasive assessment from an ECG that is often already being recorded while laboratory confirmation is pending.

Generalizability, Calibration, and Transportability

The transportability of AI-ECG - whether performance is maintained across different institutions, populations, devices, and time periods-remains one of the principal evidence gaps. Only 15 of the 33 included primary AI/ML studies reached external, temporal, or prospective validation, or provided clinical-monitoring, utility, or implementation (10 external or temporal, three prospective, and two clinical-monitoring/utility or interventional studies), leaving most performance estimates based on internal or development cohorts. Although several models maintained good performance during external validation, others showed reduced accuracy across institutions and recording modalities, particularly with single-lead ECGs [18,23,35].

These findings are consistent with the broader AI literature, in which deep-learning models may learn dataset-specific rather than disease-specific features, limiting generalizability across sites [42]. Calibration-whether predicted probabilities or estimated potassium values correspond to observed outcomes-was rarely reported, despite its importance for clinical decision-making. A model may therefore discriminate well yet remain poorly calibrated at treatment thresholds [43]. Because the clinical consequences of false-positive and false-negative results differ across emergency, dialysis, intensive care, inpatient, and remote-monitoring settings, setting-specific external validation and recalibration should be considered essential before routine clinical deployment.

Methodological Quality and Risk of Bias

QUADAS-2 assessment placed overall risk of bias as low in only four studies, unclear in 23, and high in six, with applicability concern low in 18, unclear in 10, and high in five. Most unclear ratings reflected incomplete reporting rather than demonstrated methodological flaws, although they nevertheless reduce confidence in the generalizability of the findings. The recurring concerns - case-control enrichment, artificial class balancing, incompletely reported ECG-to-laboratory timing, unclear train-test split integrity, limited threshold prespecification, and sparse calibration - are precisely the features that inflate apparent performance. These methodological features likely explain why several small studies reported near-perfect accuracy (approximately 96%-98%) using balanced or augmented datasets [11,19,20,25,30], whereas larger, clinically representative cohorts reported more modest discrimination (AUROC approximately 0.85-0.92), suggesting that the higher figures largely reflect favorable development conditions rather than superior clinical accuracy. One study additionally carried an editorial expression of concern about data availability [18,41]; this was weighed during risk-of-bias interpretation rather than treated as grounds for exclusion, and the review's conclusions were likewise unchanged when it was omitted.

Relationship to a Prior Electrolyte-Focused Review

A recent systematic review and meta-analysis, currently available as a preprint from 2025, evaluated AI-ECG for multiple electrolyte disturbances including potassium, sodium, and calcium and reported encouraging pooled diagnostic performance for hyperkalemia and hypokalemia while identifying patient-selection bias as the principal methodological concern [5]. Our findings are broadly consistent with and complement that review but extend the evidence in several important ways. Unlike the earlier electrolyte-focused synthesis, our review specifically evaluates AI-ECG across four clinically relevant potassium tasks-hyperkalemia detection, hypokalemia detection, categorical potassium-status classification, and continuous potassium estimation. Furthermore, whereas the earlier review searched the literature through late 2024, our review extends to mid-2026. Because AI-enabled healthcare is evolving rapidly, extending the search from late 2024 to mid-2026 captured several important studies, including the first pragmatic randomized clinical trial, wearable ECG validation, serial bloodless monitoring, and additional external validation studies. Finally, rather than focusing primarily on pooled diagnostic accuracy, we framed the evidence around clinical translation and implementation readiness, with particular emphasis on validation maturity, calibration, transportability, dataset overlap, model lineage, and prevalence-dependent predictive values.

From Diagnostic Accuracy to Clinical Impact

Diagnostic accuracy is necessary but not sufficient; what ultimately matters is whether AI-ECG changes care safely and beneficially. Only two included studies moved beyond diagnostic performance toward clinical monitoring, utility, or interventional evidence [26,28]. The single pragmatic randomized trial provides important insights [28]. At the selected alert threshold, the system achieved high specificity (approximately 99%) but only moderate sensitivity (52.2% for hyperkalemia and 34.7% for hypokalemia). Although the overall rate of hyperkalemia-directed treatment did not differ significantly between study arms (8.0% vs. 7.7%), treatment within three hours increased from 41.6% to 69.1% (hazard ratio 2.23, 95% CI 1.44-3.46) among patients correctly flagged as hyperkalemic. These findings suggest that alert threshold selection and implementation strategy, rather than diagnostic discrimination alone, determine the real-world clinical impact of AI-ECG. A second study explored serial bloodless monitoring during treatment of severe hyperkalemia, showing that AI-derived potassium estimates tracked laboratory values (Bland-Altman mean difference -0.21 mmol/L), although specificity remained modest (61.8%) and patient outcomes were not evaluated [26].

These signals also indicate where AI-ECG might plausibly help first. In dialysis and end-stage renal disease, where hyperkalemia is common (raising positive predictive value) and recurrent, wearable and smartwatch ECG offered a route to ambulatory surveillance [23,25], and patient-specific or repeated-visit personalization further improved accuracy in selected models [15,40]. Conversely, widespread deployment in low-prevalence populations could generate excessive false-positive alerts, leading to alert fatigue, unnecessary investigations, or inappropriate treatment escalation, whereas false-negative results may delay recognition of clinically significant dyskalemia [44]. Because these trade-offs vary across emergency departments, dialysis units, intensive care, inpatient wards, and home monitoring, future implementation studies should evaluate treatment timing, adverse events, clinician override behaviour, workflow burden, and patient outcomes alongside diagnostic accuracy. Overall, the available evidence supports further prospective implementation evaluation of AI-ECG as a potential rule-out, triage, or monitoring adjunct for potassium disorders. It does not yet establish readiness for routine clinical deployment or support replacement of laboratory potassium measurement.

Implications for Practice and Research

For clinical practice, the current evidence supports cautious, setting-specific use of AI-ECG as an adjunctive tool for triage and monitoring rather than as a replacement for laboratory potassium measurement. Its potential clinical role may differ by setting: AI-ECG may support rule-out and triage, while higher-prevalence populations, such as patients with chronic kidney disease or those receiving dialysis, may offer more favorable positive predictive value and monitoring yield. Laboratory confirmation remains essential before potassium-directed treatment. For research, future studies should prioritize prospective multicentre validation in intended-use populations, head-to-head comparison with point-of-care testing, robust external validation and calibration, standardized reporting in accordance with TRIPOD+AI [45] and DECIDE-AI [46], and evaluation of clinically meaningful outcomes alongside diagnostic accuracy. Studies should also clearly distinguish patient-level from beat- or segment-level analyses and explicitly report dataset overlap and model lineage to improve transparency and reproducibility.

Limitations

This review has several limitations. First, the available evidence was highly heterogeneous with respect to potassium targets, diagnostic thresholds, ECG modalities, model architectures, validation strategies, reporting units, and performance metrics, precluding quantitative synthesis. Second, many included studies had unclear or high risk of bias, with limited reporting of calibration, confidence intervals, and other methodological details. Third, some performance measures were derived from published figures, tables, or confusion matrices when not explicitly reported by the authors. Fourth, several studies reported selected operating points or non-standard performance metrics, limiting direct comparison across clinically relevant thresholds. Fifth, we did not directly search Scopus, Web of Science, Embase, or IEEE Xplore because these sources were not accessible to the review team. We attempted to mitigate this limitation through searches of PubMed/MEDLINE, Europe PMC, OpenAlex, Semantic Scholar, and the Cochrane Library/CENTRAL, together with backward and forward citation chasing and targeted publisher, repository, and bibliographic checks. These supplementary methods may have reduced, but cannot eliminate, the risk that eligible studies were missed. Finally, the review was not prospectively registered; some overlapping datasets could not be completely verified despite detailed lineage assessment, and although study selection, data extraction, and risk-of-bias assessment were independently verified by additional reviewers, reviewer-related bias cannot be entirely excluded. These limitations should be considered when interpreting the findings but do not alter the overall conclusion that AI-ECG shows considerable promise while requiring further high-quality prospective validation before routine clinical implementation.

## Conclusions

AI-enabled ECG represents a promising but still developing approach for potassium assessment. Current evidence was strongest for hyperkalemia detection, whereas hypokalemia detection, potassium-status classification, and continuous potassium estimation remain comparatively less mature. Although many studies reported good diagnostic discrimination, the evidence was dominated by retrospective development and internal-validation designs, with relatively few studies progressing to prospective or clinical-utility evaluation. AI-ECG should therefore be regarded as an adjunctive tool to support triage and monitoring rather than a replacement for laboratory potassium measurement. Future research should prioritize prospective multicentre validation, standardized reporting, calibration, reproducibility, and evaluation of patient-centered and workflow outcomes to establish clinical effectiveness.

## Appendices

Appendix 1: Search methodology and information sources

**Table 4:** Search methodology and information sources ANFIS: adaptive neuro-fuzzy inference system; DOI: digital object identifier; PRISMA-S: Preferred Reporting Items for Systematic Reviews and Meta-Analyses Search Extension

Item	Details
Reporting guideline	Search strategy reported in accordance with PRISMA-S.
Search period	Searches conducted between April and June 2026, with a final update on June 18, 2026.
Language restrictions	None applied during searching.
Eligibility period	Studies published between 2016 and 2026.
Core biomedical literature sources	PubMed/MEDLINE, Europe PMC
Supplementary discovery and publication-status sources	OpenAlex, Semantic Scholar, ClinicalTrials.gov, medRxiv, targeted arXiv and repository searches, and the Cochrane Library/CENTRAL.
Bibliographic verification	Crossref was used for DOI and bibliographic verification.
Additional discovery and verification methods	Backward and forward citation chasing and record-specific checks of publisher pages, repositories, and engineering metadata sources, including DBLP, IEEE metadata or DOI pages, KCI, ScienceON, RISS, OpenReview, and institutional repositories, were used for bibliographic, publication-status, and publication-lineage verification. These were targeted supplementary checks rather than comprehensive formal searches of each source.
Search adaptation	Database-specific search strategies were adapted while preserving the core concepts of potassium abnormalities, electrocardiography, and artificial intelligence/machine learning.
Preprints and non-primary records	Considered during study identification but excluded from the primary evidence synthesis unless linked to an eligible peer-reviewed publication.
Search strategy	Appendix 2 presents the principal formal source-specific queries used in PubMed/MEDLINE, Europe PMC, ClinicalTrials.gov, the Cochrane Library/CENTRAL, medRxiv, OpenAlex, and Semantic Scholar. Other sources were used for targeted citation, bibliographic, publication-status, or lineage verification and are described narratively in Appendix 1 rather than represented as comprehensive formal search strategies.

Appendix 2: Source-specific search strategies

**Table 5:** Search strategies used AI: artificial intelligence; CENTRAL: Cochrane Central Register of Controlled Trials; ECG: electrocardiogram/electrocardiography; EKG: electrocardiogram; MeSH: Medical Subject Headings; tiab: title and abstract; TITLE_ABS: title and abstract search field. An asterisk (*) indicates truncation where supported by the respective platform.

Source	Source-specific queries and the terms used
PubMed/MEDLINE	(("Hyperkalemia"[Mesh] OR "Hypokalemia"[Mesh] OR hyperkalem*[tiab] OR hyperkalaem*[tiab] OR hypokalem*[tiab] OR hypokalaem*[tiab] OR dyskalem*[tiab] OR dyskalaem*[tiab] OR potassium[tiab] OR "serum potassium"[tiab] OR "plasma potassium"[tiab] OR (electrolyte[tiab] AND (imbalance*[tiab] OR abnormalit*[tiab] OR disorder*[tiab] OR disturbance*[tiab]))) AND ("Electrocardiography"[Mesh] OR "Electrocardiography, Ambulatory"[Mesh] OR ECG[tiab] OR EKG[tiab] OR electrocardiogram*[tiab] OR electrocardiograph*[tiab] OR electrocardiographic[tiab] OR "12-lead"[tiab] OR "12 lead"[tiab] OR "single-lead"[tiab] OR "single lead"[tiab] OR smartwatch*[tiab] OR wearable*[tiab] OR Holter[tiab]) AND ("Artificial Intelligence"[Mesh] OR "Machine Learning"[Mesh] OR "artificial intelligence"[tiab] OR "machine learning"[tiab] OR "deep learning"[tiab] OR "neural network"[tiab] OR "neural networks"[tiab] OR algorithm*[tiab] OR (predict*[tiab] AND model*[tiab]) OR convolutional[tiab] OR transformer*[tiab]))
Europe PMC	((TITLE_ABS:hyperkalem* OR TITLE_ABS:hyperkalaem* OR TITLE_ABS:hypokalem* OR TITLE_ABS:hypokalaem* OR TITLE_ABS:dyskalem* OR TITLE_ABS:dyskalaem* OR TITLE_ABS:potassium OR TITLE_ABS:"serum potassium" OR TITLE_ABS:"plasma potassium" OR (TITLE_ABS:electrolyte AND (TITLE_ABS:imbalance* OR TITLE_ABS:abnormalit* OR TITLE_ABS:disorder* OR TITLE_ABS:disturbance*))) AND (TITLE_ABS:ECG OR TITLE_ABS:EKG OR TITLE_ABS:electrocardiogram* OR TITLE_ABS:electrocardiograph* OR TITLE_ABS:electrocardiographic OR TITLE_ABS:"12-lead" OR TITLE_ABS:"12 lead" OR TITLE_ABS:"single-lead" OR TITLE_ABS:"single lead" OR TITLE_ABS:smartwatch* OR TITLE_ABS:wearable* OR TITLE_ABS:Holter) AND (TITLE_ABS:"artificial intelligence" OR TITLE_ABS:"machine learning" OR TITLE_ABS:"deep learning" OR TITLE_ABS:"neural network" OR TITLE_ABS:"neural networks" OR TITLE_ABS:algorithm* OR (TITLE_ABS:predict* AND TITLE_ABS:model*) OR TITLE_ABS:convolutional OR TITLE_ABS:transformer*))
ClinicalTrials.gov	(ECG OR electrocardiogram OR EKG OR single-lead OR wearable) AND (potassium OR hyperkalemia OR hypokalemia OR dyskalemia OR electrolyte) AND ("artificial intelligence" OR "machine learning" OR "deep learning" OR algorithm OR prediction)
Semantic Scholar	AI ECG potassium hyperkalemia hypokalemia dyskalemia electrolyte estimation machine learning deep learning
OpenAlex	AI ECG potassium hyperkalemia hypokalemia dyskalemia electrolyte estimation machine learning deep learning
Cochrane Library/CENTRAL	(ECG OR electrocardiogram OR EKG) AND (potassium OR hyperkalemia OR hypokalemia OR dyskalemia) AND ("artificial intelligence" OR "machine learning" OR "deep learning" OR algorithm)
medRxiv	(potassium OR hyperkalemia OR hypokalemia OR dyskalemia) AND (ECG OR EKG OR electrocardiogram OR electrocardiography) AND ("artificial intelligence" OR "machine learning" OR "deep learning" OR algorithm)

Appendix 3: Supplementary results

**Table 6:** Per-study characteristics, AI/ML methods, validation approach, and key findings of the 33 included primary studies Data extracted from the included primary AI/ML studies [3,4,10-40]. Metrics are reported as presented in the original studies or derived where indicated. Classification and regression outcomes were analysed separately and were not pooled. AI: artificial intelligence; AUROC: area under the receiver operating characteristic curve; CKD: chronic kidney disease; CNN: convolutional neural network; CRNN: convolutional recurrent neural network; DWT: discrete wavelet transform; ECG: electrocardiography; ED: emergency department; EMD: empirical mode decomposition; ESRD: end-stage renal disease; FCM-ANFIS: fuzzy c-means adaptive neuro-fuzzy inference system; FIS: fuzzy inference system; ICU: intensive care unit; kNN: k-nearest neighbors; MAE: mean absolute error; ML: machine learning; NPV: negative predictive value; PPV: positive predictive value; RCT: randomized controlled trial; SVM: support vector machine; TPP: thyrotoxic periodic paralysis; CI: Confidence Interval

Study	Setting/population	ECG input	Potassium task	AI/ML method and validation	Key findings and limitations
Galloway et al., 2019 [3]	USA Mayo Clinic; patients with CKD stage >= 3; validation n=61,965.	Standard 12-lead ECG; reduced 2-lead or 4-lead input derived from 12-lead ECG.	Hyperkalemia, mainly K+ >=5.5 mmol/L.	Deep CNN; retrospective multi-site validation.	AUROC 0.883 (95% CI 0.873-0.893), 0.860 (0.837-0.883), and 0.853 (0.830-0.877) across sites; CKD-enriched cohort, near-threshold values excluded, and PPV remained low.
Lin et al., 2020 [4]	Taiwan; Retrospective ED cohort; n=40,180 patients / 66,321 ECG-K+ pairs.	Standard 12-lead digital ECG.	Hyperkalemia, hypokalemia, categorical status, and continuous K+ estimation.	ECG12Net deep CNN; temporal validation (data split by date)	In the 300-ECG human-machine competition set, AUROC was 0.958 for hyperkalemia and 0.926 for hypokalemia. Sensitivity was 83.3% (95% CI 73.3-91.7) for severe hyperkalemia and 96.7% (95% CI 91.7-100.0) for severe hypokalemia. The study used retrospective single-centre data, and performance was weaker when mild-to-moderate dyskalemia was included, limiting generalizability beyond severe abnormalities.
Kwon et al., 2021 [10]	South Korean two-hospital electrolyte-testing cohort; n=92,140.	12-lead ECG, limb leads, and lead I inputs.	Hyperkalemia and hypokalemia, with multi-electrolyte outputs.	Residual CNN with age and sex features; internal and external validation.	External AUROC: 0.873 (95% CI 0.843-0.902) for hyperkalemia and 0.857 (0.846-0.867) for hypokalemia; low abnormality prevalence caused very low PPV.
Taguelmimt et al., 2022 [11]	France; hemodialysis/healthy-control dataset; 736 ECG images.	ECG beat images derived from ECG recordings.	Hyperkalemia severity classification.	CNN image classifier; development-only proof of concept.	Test accuracy 96.08% with sensitivity 97.89%; very small dataset, synthetic classes, and beat-level non-independence limit clinical inference.
Urtnasan et al., 2022 [12]	South Korea; emergency cohort with chronic renal failure enrichment; n=855 (555 with CKD; 300 without)	Single-lead segments derived from 12-lead ECG.	Hyperkalemia, K+ >5.5 mmol/L.	Lightweight 1D CNN; internal validation.	Lead II test F1 score 0.95; retrospective single-center case-control design with no external validation.
Lin et al., 2022 [13]	Taiwan; two-hospital ED cohorts; 31,246 patients / 41,295 visits.	Standard 12-lead ECG with AI-estimated ECG-K+.	Hyperkalemia, hypokalemia, categorical status, and continuous K+ estimation.	ECG12Net; multi-site validation with silent real-time calculation.	MAE about 0.364-0.365 mmol/L and severe hyperkalemia AUROC 0.966-0.987; low PPV and no clinician-facing intervention.
Moon et al., 2022 [14]	South Korea; hospital ECG-laboratory dataset; n=1,879.	Precordial-lead V1-V6; ECG cycles, mainly V5.	Continuous K+ estimation and hyperkalemia monitoring.	Lightweight CRNN; internal validation.	Best-performing lead R2=0.745 and MAE=0.450 mEq/L; single-center study without diagnostic AUROC or external validation.
Lou et al., 2023 [15]	Taiwan; repeated-visit cohorts; 168,450 development ECG-K+ pairs.	Standard 12-lead ECG.	Hyperkalemia, hypokalemia, and continuous K+ estimation.	Deep ECG model plus dynamic personal revision; external validation.	Dynamic revision AUROC 0.915-0.919 for hyperkalemia; requires prior patient ECG-K+ pairs and hypokalemia AUROC remained below 0.8.
Kim et al., 2023 [16]	South Korea; ED case-control cohort; n=125.	Smartphone-captured 12-lead ECG images.	Hyperkalemia, K+ >=6.0 mmol/L.	Smartphone-based deep learning ECG Image analyser app; internal diagnostic validation.	App AUROCs ranged 0.898 (95% CI 0.842-0.954) to 0.904 (0.849-0.959). The small enriched case-control cohort (n=125), internal-only validation, and absence of clinical-outcome or workflow evaluation limit generalizability to routine emergency-department populations.
Xu et al., 2023 [17]	China; maintenance hemodialysis ESRD cohort; n=80 / 1,024 paired datasets.	12-lead ECG with engineered chest-lead features.	Hyperkalemia at multiple thresholds from K+ >=5.0 to >=6.5 mmol/L.	Feature-based ML models including XGBoost, SVM, CNN, and AdaBoost; internal validation.	XGBoost AUROC 0.931 (95% CI 0.912-0.953) at K+ >=5.0 and 0.872 (0.843-0.906) at >=5.5; small repeated dialysis dataset and worse performance at severe thresholds.
An et al., 2024 [18]	South Korea; two-hospital adult cohorts; external validation 130,415 ECG-K+ pairs.	12-lead, limb-lead, and lead I ECG.	Hyperkalemia and hypokalemia.	SE-ResNet deep learning; external validation.	External AUROC 0.923 (95% CI 0.914-0.931) for hyperkalemia and 0.913 (0.910-0.916) for hypokalemia; retrospective design, low PPV, and later expression of concern about data availability.
Thomas et al., 2024 [19]	MIMIC-III ICU database	20 second-ECG waveform segments with extracted signal features.	Hyperkalemia.	ANN and SVM classifiers; development-only analysis.	Reported ANN AUROC 0.994 and SVM AUROC 0.985; segment-level analysis with limited clinical detail and no external prospective validation.
Varghese, 2024 [20]	MIMIC-IV-ECG Diagnostic Electrocardiogram Matched Subset linked to the MIMIC-IV clinical database; 1,790 ECG–potassium pairs; ECG and serum-potassium measurements were obtained less than four hours apart.	ECG morphology and empirical-mode-decomposition features.	Hyperkalemia, defined as serum K+ >6.0 mEq/L.	AdaBoost, kernel naïve Bayes, and neural-network classifiers; development-only internal evaluation	AdaBoost using morphological and empirical-mode-decomposition features achieved sensitivity of 97.7%, specificity of 98.84%, and accuracy of 98.3%. Artificial class balancing, inconsistent reporting of the held-out test proportion, absence of external validation, and limited reporting of clinically representative predictive values restrict generalizability.
Bergquist et al., 2024 [21]	USA; University of Utah adult health-system data.	12-lead ECG.	Hyperkalemia prediction at different ECG-lab time cutoffs.	Machine-learning classifiers; internal validation.	AUROC 0.850 at 1 hour and 0.814 at 15 minutes; conference-style report and no external validation.
Samandari et al., 2024 [22]	South Korea; ECG-ViEW II public database; selected patient dataset; n=42.	ECG interval, axis, and derived features.	Hyperkalemia, hypokalemia, categorical status, and continuous K+ estimation.	FCM-ANFIS and feature-based models; internal validation.	Hypokalemia sensitivity 100% and hyperkalemia sensitivity 80%; feature-selected high-risk dataset limits applicability.
Chiu et al., 2024 [23]	USA+Taiwan; ESRD cohorts; training n=152,508, external ESRD n=3,107, smartwatch n=40.	12-lead ECG and single-lead Apple Watch ECG.	Hyperkalemia detection and continuous K+ estimation.	Kardio-Net deep CNN; external and prospective device validation.	12-lead AUROC 0.852 (95% CI 0.745-0.956) primary and 0.849 (0.823-0.875) external; smartwatch AUROC 0.831 (0.693-0.975); severe hyperkalemia cases were few and no intervention was tested.
Harmon et al., 2024 [24]	USA; ED and ICU validation cohorts; ED n=40,128, ICU n=2,636.	Standard 12-lead ECG analyzed using leads I and II.	Hyperkalemia, primarily K+ >6.0 mmol/L.	Previously developed CNN AI-ECG model; independent retrospective validation.	At K+ >6.0, AUROC 0.88 (95% CI 0.86-0.90) in ED and 0.88 (0.83-0.92) in ICU; very low PPV, especially in the ED, limits rule-in use.
Shang et al., 2025 [25]	Dialysis patients with stage-5 CKD; n=33.	Wearable single-lead ECG from chest strap device.	Hyperkalemia.	1D CNN monitoring system; development-only validation.	Reported sensitivity 98.63% and specificity 97.88%; limited cohort detail and no independent prospective deployment.
Chen et al., 2025 [26]	Taiwan; emergency severe-hyperkalemia treatment cohort; n=76.	AI-enabled ECG potassium estimation.	Hyperkalemia monitoring.	AI-ECG potassium model; clinical-monitoring study.	Severe-hyperkalemia monitoring sensitivity/specificity 86.7%/61.8%; not an interventional trial and specificity was modest.
Lin et al., 2025 [27]	Taiwan; ICU diagnostic-comparison cohort; n=267.	Standard 12-lead ECG.	Hyperkalemia, K+ ≥ 5.5 mEq/L.	Previously developed AI-ECG potassium model; internal diagnostic comparison.	AI-ECG AUROC 0.884 and NPV 99.4%; only 15 hyperkalemia cases and PPV was 13.2%.
Lin et al., 2026 [28]	Taiwan; ED pragmatic, open-label, randomized controlled trial; 70 ED physicians / 14,989 patients.	12-lead ECG; AI-enabled ECG alert.	Hyperkalemia and hypokalemia alerts.	AI-ECG alert system; clinical-utility and interventional evidence.	Diagnostic AUROC 0.971 for hyperkalemia and 0.906 for hypokalemia; no significant overall treatment increase within 3 hours.
Iyigun et al., 2026 [29]	Turkish multicenter heart-failure cohort with LVEF <=45%.	Clinical ECG images with lead D2 beat segments.	Hyperkalemia, hypokalemia, and hyponatremia.	CNN model; prospective multicenter collection with internal validation.	AUROC 0.94 (95% CI 0.87-0.98) for hyperkalemia and 0.84 (0.76-0.91) for hypokalemia; potassium cutoffs and reporting were partly unclear.
Abassi et al., 2026 [30]	Tunisia; Secondary ECG12Net/competition-derived dataset; 300 ECG recordings.	Raw 12-lead ECG with wavelet-derived features.	Five-class hyperkalemia and hypokalemia classification.	SVM, Random Forest, kNN, AlexNet, and CNN-LSTM; development-only benchmark.	Best CNN-LSTM plus DWT accuracy 96%; small balanced augmented dataset may inflate performance.
Lee et al., 2026 [31]	USA Mayo Clinic Platform emergency-diagnosis cohort; n=3,740 encounters.	Rendered 12-lead ECG image.	Hyperkalemia, K+ >=5.5 mmol/L.	Image-based deep learning biomarker; large external validation.	AUROC 0.917 (95% CI 0.908-0.926), sensitivity 83.8% (95% CI 81.8-85.8), and specificity 83.8% (95% CI 82.4-85.3); case-enriched sampling limits real-world PPV and NPV.
Dade et al., 2026 [32]	USA; University of Utah ECG pretraining dataset; 29,695 patients / about 59,000 ECGs.	Eight independent leads from 12-lead ECG.	High serum potassium, >5.0 mEq/L; the source article labels the downstream laboratory target as potassium chloride (KCl).	Self-supervised contrastive ECG representation learning; development-only benchmark.	Pretraining improved KCl AUROC by about 5-7 points in low-label settings; standalone clinical metrics were not tabulated.
Kumar et al., 2026 [33]	India single tertiary ED/cardiology clinic; prospective n=4,200.	Standard 12-lead ECG with filtered and segmented signal windows.	Hyperkalemia, K+ >=5.5 mmol/L; critical K+ >=6.0.	CNN-based AI-ECG system; prospective single-center clinical evaluation.	AUROC 0.901 (95% CI 0.884-0.918), sensitivity 83.5% (95% CI 80.2-86.4), specificity 87.3% (95% CI 85.9-88.6), and median time-to-therapy 21 vs 40 minutes; model provenance was incompletely described and no external validation was reported.
Lin et al., 2021 [34]	Taiwan; male patients with acute weakness/ hypokalemic paralysis cohorts; development 31 TPP/414 controls, prospective n=22.	Standard 12-lead ECG with ECG-K+ and ECG-TPP outputs.	Hypokalemia, Continuous K+ estimation and thyrotoxic periodic paralysis, K+ <=3.0 mEq/L.	ECG12Net-derived models; small prospective feasibility validation.	Integrated AI plus laboratory model reached AUC 0.986 and prospective PPV 100%; TPP-specific, male-only, and very small.
Wang et al., 2021 [35]	China; emergency patients with external branch-hospital validation; 9,908 ECGs.	Standard 12-lead ECG and single-lead lead II model.	Hypokalemia, K+ <3.5 mmol/L.	11-layer CNN; external validation.	AUROC 0.80 (95% CI 0.77-0.82) internal and 0.77 (0.75-0.79) external; balanced sampling and weak single-lead performance limit deployment readiness.
Wang et al., 2022 [36]	China; single-hospital ECG-serum potassium dataset; 2,760 ECG-K+ pairs.	Standard 10-second 12-lead ECG.	Hypokalemia, K+ <3.5 mmol/L.	Two-stream CNN combining raw ECG and T-wave features; internal validation.	Test AUROC 0.82 with sensitivity 77.54% and specificity 74.28%; single-center balanced data and near-normal samples excluded.
von Bachmann et al., 2024 [37]	Swedish multicenter ED electrolyte dataset; 165,508 patients / 290,889 ECGs.	Eight independent leads from standard 12-lead ECG.	Continuous K+ estimation plus hyperkalemia and hypokalemia classification.	ResNet-based deep regression and probabilistic models; temporal validation.	MAE 0.285 mmol/L random test and 0.262 temporal test; no external geographic validation and calibration remained imperfect.
Babur et al., 2024 [38]	MIMIC-III ICU waveform/clinical records; 116 potassium records.	Lead II ECG features.	Continuous potassium and calcium estimation.	Neural-network regression and fuzzy inference system; internal hold-out validation.	Potassium FIS validation MSE 0.3162 with R 0.607; only 116 potassium records and NN regression overfit.
Nowroozilarki et al., 2024 [39]	MIMIC-IV ECG and clinical electrolyte data.	12-lead ECG, mainly lead II for downstream prediction.	Continuous K+ estimation with dyskalemia classification.	ECG2Electrolyte semi-supervised contrastive learning; development-only benchmark.	Potassium MAE 0.49 and macro AUROC 0.69; benchmark-stage MIMIC-IV data, 3-hour ECG-lab pairing, and modest discrimination.
Chiu et al., 2022 [40]	MIMIC-III ICU waveform dataset; generic n=1,439, personalized n=16.	Single-lead monitor ECG, mainly lead II.	Hyperkalemia, K+ >5.5 mEq/L.	1D ResNet-50 with personalized transfer learning; internal validation.	Personalized AUC improved from 0.729 to 0.945; only 16 personalized patients and prior patient-specific labels were required.

## References

[1] Kim MJ, Valerio C, Knobloch GK (2023). Potassium disorders: hypokalemia and hyperkalemia. Am Fam Physician.

[2] Pilia N, Severi S, Raimann JG (2020). Quantification and classification of potassium and calcium disorders with the electrocardiogram: what do clinical studies, modeling, and reconstruction tell us?. APL Bioeng.

[3] Galloway CD, Valys AV, Shreibati JB (2019). Development and validation of a deep-learning model to screen for hyperkalemia from the electrocardiogram. JAMA Cardiol.

[4] Lin CS, Lin C, Fang WH (2020). A deep-learning algorithm (ECG12Net) for detecting hypokalemia and hyperkalemia by electrocardiography: algorithm development. JMIR Med Inform.

[5] Dasdelen MF, Almas F, Dasdelen ZB, Yapalak ANB, Marr C (2025). AI in ECG-based electrolyte imbalance prediction: a systematic review and meta-analysis (Preprint).

[6] McInnes MD, Moher D, Thombs BD (2018). Preferred reporting items for a systematic review and meta-analysis of diagnostic test accuracy studies: the PRISMA-DTA statement. JAMA.

[7] Page MJ, McKenzie JE, Bossuyt PM (2021). The PRISMA 2020 statement: an updated guideline for reporting systematic reviews. BMJ.

[8] Rethlefsen ML, Kirtley S, Waffenschmidt S, Ayala AP, Moher D, Page MJ, Koffel JB (2021). PRISMA-S: an extension to the PRISMA statement for reporting literature searches in systematic reviews. Syst Rev.

[9] Whiting PF, Rutjes AW, Westwood ME (2011). QUADAS-2: a revised tool for the quality assessment of diagnostic accuracy studies. Ann Intern Med.

[10] Kwon JM, Jung MS, Kim KH (2021). Artificial intelligence for detecting electrolyte imbalance using electrocardiography. Ann Noninvasive Electrocardiol.

[11] Taguelmimt K, Wojak J, Adel M (2022). Blood potassium concentration estimation on electrocardiogram signals using machine learning techniques. 2022 7th International Conference on Frontiers of Signal Processing (ICFSP).

[12] Urtnasan E, Lee JH, Moon B, Lee HY, Lee K, Youk H (2022). Noninvasive screening tool for hyperkalemia using a single-lead electrocardiogram and deep learning: development and usability study. JMIR Med Inform.

[13] Lin C, Chau T, Lin CS (2022). Point-of-care artificial intelligence-enabled ECG for dyskalemia: a retrospective cohort analysis for accuracy and outcome prediction. NPJ Digit Med.

[14] Moon B, Byun J, Park Y, Youk H, Lee H, Choo Y (2022). Screening of ECG change with serum potassium level based on deep learning for monitoring hyperkalemia (Article in Japanese). J Inst Electron Inf Eng.

[15] Lou YS, Lin CS, Fang WH, Lee CC, Wang CH, Lin C (2023). Development and validation of a dynamic deep learning algorithm using electrocardiogram to predict dyskalaemias in patients with multiple visits. Eur Heart J Digit Health.

[16] Kim D, Jeong J, Kim J (2023). Hyperkalemia detection in emergency departments using initial ECGs: a smartphone AI ECG analyzer vs. board-certified physicians. J Korean Med Sci.

[17] Xu D, Zhou B, Zhang J (2023). Prediction of hyperkalemia in ESRD patients by identification of multiple leads and multiple features on ECG. Ren Fail.

[18] An JN, Park M, Joo S (2024). Development of deep learning algorithm for detecting dyskalemia based on electrocardiogram. Sci Rep.

[19] Thomas A, Lokulwar P, Bora V (2024). ECG signal classification for detection of hyperkalemia. J Electr Syst.

[20] Varghese AT (2024). Integrating morphological characteristics with empirical mode decomposition for robust ECG signal classification. Commun Appl Nonlinear Anal.

[21] Bergquist JA, Dade D, Zenger B (2024). Machine learning prediction of blood potassium at different time cutoffs. Comput Cardiol (2010).

[22] Samandari Z, Molaeezadeh SF (2024). Noninvasive estimation of blood potassium concentration using ECG and FCM-ANFIS model. Res Biomed Eng.

[23] Chiu IM, Wu PJ, Zhang H (2024). Serum potassium monitoring using AI-enabled smartwatch electrocardiograms. JACC Clin Electrophysiol.

[24] Harmon DM, Liu K, Dugan J, Jentzer JC, Attia ZI, Friedman PA, Dillon JJ (2024). Validation of noninvasive detection of hyperkalemia by artificial intelligence-enhanced electrocardiography in high acuity settings. Clin J Am Soc Nephrol.

[25] Shang H, Yu S, Wu Y (2025). A noninvasive hyperkalemia monitoring system for dialysis patients based on a 1D-CNN model and single-lead ECG from wearable devices. Sci Rep.

[26] Chen CC, Lin C, Lee DJ (2025). Monitoring serum potassium concentration in patients with severe hyperkalemia: the role of bloodless artificial intelligence-enabled electrocardiography. Clin Kidney J.

[27] Lin C, Chen CC, Lin CS, Shang HS, Lee CC, Chau T, Lin SH (2025). Point-of-care potassium measurement vs artificial intelligence-enabled electrocardiography for hyperkalemia detection. Am J Crit Care.

[28] Lin C, Lin CS, Chen SJ (2026). AI-enabled electrocardiogram alert for potassium imbalance treatment: a pragmatic randomized controlled trial. Nat Commun.

[29] İyigün U, Kerkütlüoğlu M, Güneş H (2026). Detection of hypokalemia, hyponatremia, and hyperkalemia in heart failure patients using artificial intelligence techniques via electrocardiography. Turk Kardiyol Dern Ars.

[30] Abassi S, Mahersia H, Elhechmi YZ (2026). Dyskalemia detection based on wavelet scattering features and deep learning models: a comparative analysis. Biomed Signal Process Control.

[31] Lee H, Kim Y, Sykora D, Ryu AJ, Cho Y, Kim J, Song J (2026). External validation of ECG artificial intelligence for emergency and cardiac assessment across a large-scale U.S. healthcare system. NPJ Digit Med.

[32] Dade D, Bergquist JA, MacLeod RS, Steinberg BA, Tasdizen T (2026). Self-supervised contrastive learning enables robust electrocardiogram-based cardiac classification. Heart Rhythm O2.

[33] Kumar A, Bansal T, Jaura S (2026). AI-powered ECG interpretation system for arrhythmia prediction and hyperkalemia detection using machine learning. J Assoc Physicians India.

[34] Lin C, Lin CS, Lee DJ (2021). Artificial intelligence-assisted electrocardiography for early diagnosis of thyrotoxic periodic paralysis. J Endocr Soc.

[35] Wang CX, Zhang YC, Kong QL (2021). Development and validation of a deep learning model to screen hypokalemia from electrocardiogram in emergency patients. Chin Med J (Engl).

[36] Wang Y, Zhong G, Wang Y (2022). Intelligent detection of hypokalemia based on 12-lead ECG using two-stream deep learning model. 2022 15th International Congress on Image and Signal Processing, BioMedical Engineering and Informatics (CISP-BMEI).

[37] von Bachmann P, Gedon D, Gustafsson FK (2024). Evaluating regression and probabilistic methods for ECG-based electrolyte prediction. Sci Rep.

[38] Babur S, Moghaddamnia S, Bozkurt MR (2024). Estimation of blood calcium and potassium values from ECG records. Meas Sci Rev.

[39] Nowroozilarki Z, Huang S, Khera R, Mortazavi BJ (2024). Non-invasive electrolyte estimation using multi-lead ECG data via semi-supervised contrastive learning with an adaptive loss. 2024 IEEE EMBS International Conference on Biomedical and Health Informatics (BHI).

[40] Chiu IM, Cheng JY, Chen TY (2022). Using deep transfer learning to detect hyperkalemia from ambulatory electrocardiogram monitors in intensive care units: personalized medicine approach. J Med Internet Res.

[41] An JN, Park M, Joo S (2025). Editorial expression of concern: development of deep learning algorithm for detecting dyskalemia based on electrocardiogram. Sci Rep.

[42] Zech JR, Badgeley MA, Liu M, Costa AB, Titano JJ, Oermann EK (2018). Variable generalization performance of a deep learning model to detect pneumonia in chest radiographs: a cross-sectional study. PLoS Med.

[43] Van Calster B, McLernon DJ, van Smeden M, Wynants L, Steyerberg EW (2019). Calibration: the Achilles heel of predictive analytics. BMC Med.

[44] Ancker JS, Edwards A, Nosal S, Hauser D, Mauer E, Kaushal R (2017). Effects of workload, work complexity, and repeated alerts on alert fatigue in a clinical decision support system. BMC Med Inform Decis Mak.

[45] Collins GS, Moons KG, Dhiman P (2024). TRIPOD+AI statement: updated guidance for reporting clinical prediction models that use regression or machine learning methods. BMJ.

[46] Vasey B, Nagendran M, Campbell B (2022). Reporting guideline for the early-stage clinical evaluation of decision support systems driven by artificial intelligence: DECIDE-AI. Nat Med.

